# Identification of critical links in a large-scale road network considering the traffic flow betweenness index

**DOI:** 10.1371/journal.pone.0227474

**Published:** 2020-04-10

**Authors:** Feiyan Li, Hongfei Jia, Qingyu Luo, Yongxing Li, Lili Yang

**Affiliations:** 1 College of Transportation, Jilin University, Changchun, Jilin, China; 2 Rail Transit Institute, Jilin Communications Polytechnic, Changchun, Jilin, China; 3 School of Civil and Environmental Engineering, Nanyang Technological University, Singapore, Singapore; University of British Columbia, CANADA

## Abstract

The traditional full-scan method is commonly used for identifying critical links in road networks. This method simulates each link to be closed iteratively and measures its impact on the efficiency of the whole network. It can accurately identify critical links. However, in this method, traffic assignments are conducted under all scenarios of link disruption, making this process prohibitively time-consuming for large-scale road networks. This paper proposes an approach considering the traffic flow betweenness index (*TFBI*) to identify critical links, which can significantly reduce the computational burden compared with the traditional full-scan method. The *TFBI* consists of two parts: traffic flow betweenness and endpoint origin–destination (OD) demand (rerouted travel demand). There is a weight coefficient between these two parts. Traffic flow betweenness is established by considering the shortest travel-time path betweenness, link traffic flow and total OD demand. The proposed approach consists of the following main steps. First, a sample road network is selected to calibrate the weight coefficient between traffic flow betweenness and endpoint OD demand in the *TFBI* using the network robustness index. This index calculates changes in the whole-system travel time due to each link’s closure under the traditional full-scan method. Then, candidate critical links are pre-selected according to the *TFBI* value of each link. Finally, a given number of real critical links are identified from the candidate critical links using the traditional full-scan method. The applicability and computational efficiency of the *TFBI*-based approach are demonstrated for the road network in Changchun, China.

## Introduction

Urban road networks are the lifeblood of the development of cities and significantly affect the travel of residents and the logistics of production. However, links are often interrupted by natural hazards, traffic accidents. The failure of certain critical links can significantly degrade a road network’s performance and can even trigger a cascading failure, paralysing the network for some time. Hence, identifying and enhancing critical links can avoid or mitigate the influence of failures on the network when chance events or intentional attacks occur.

Reliability, vulnerability, and criticality (critical components) are related concepts, but unified definitions of these concepts have not been universally accepted. Asakura et al. defined road network reliability as a road network’s ability to handle a recurrent variation [1]. Similarly, Berdica described road network vulnerability as “susceptibility to incidents that can result in considerable reductions in road network serviceability” [2]. As a branch of reliability analysis [3], vulnerability analysis assesses the effects of network degradation caused by disruptive events, such as adverse weather conditions, traffic accidents, and malicious attacks [4]. Criticality is defined in terms of the effects of network degradation, such as socioeconomic effects, and increases with generalized travel costs [5]. Critical components are determined with respect to the severity of disruptions in network performance when nodes or links fail [6, 7]. In this paper, link criticality emerges as a measure for assessing the negative consequences of such link interruption on the whole road network. The worse the negative consequence is, the more critical the link will be.

Numerous indicators have been proposed for identifying critical components in terms of road network efficiency changes caused by link disruption. These indicators include accessibility, travel cost, and the network robustness index (*NRI*) as well as mixed indicators. Sohn used an accessibility indicator to identify critical links in the Maryland highway network [8]. Taylor et al. presented a cost indicator considering generalized travel cost, the Hansen integral accessibility indicator [9], and the accessibility/ remoteness index of Australia (Australian *ARIA*) [6]. Jenelius et al. determined critical links based on increases in generalized travel costs due to link disruption and introduced the concept of unsatisfied demand [5]. Scott et al. presented the *NRI* considering traffic flow, link capacity and network topology to identify critical links [10]. Improved *NRI* indicators have also been proposed, such as network trip robustness (*NTR*) [11] and the network vulnerability index (*NVI*) [12]. Oliveira et al. identified critical links using congestion indicators (*V/C* and *CI*) and the *NRI* and *NTR* and found that the *NRI* and *NTR* tended to be more accurate [13]. Rupi et al. presented a mixed indicator combining a link’s level of use (how many people typically use the link) and the consequences of link closure on the whole network [14]. Du et al. proposed a capacity-based network robustness index to identify critical links [15].

Generally, most of these indicators are systematic, having been computed using the traditional full-scan method, which assesses the variations in the efficiency of the whole road network caused by each link closure. The traditional full-scan method can accurately identify critical components using many kinds of data, such as predicted link traffic flow responses to link closure based on the origin-destination (OD) demand and traffic flow assignment models. Accordingly, with each link removal, the loss of whole-system efficiency is reevaluated through executing the traffic assignment model. Consequently, calculations for large-scale road networks using the traditional full-scan method can take prohibitive amounts of time [16]. The purpose of this study is to find an approach for identifying the critical links of large-scale road networks with a lower computational burden. To our knowledge, few scholars have proposed approaches for identifying critical links in large-scale road networks. Zhang X, Guo C and Wang L proposed an approach for identifying critical components using Monte Carlo sampling and game theory [17]. Luathep P, Sumalee A, Ho HW and Kurauchi F presented a sensitivity-analysis-based approach to improve computational efficiency when handling a large-scale road network [18]. Chen BY, Lam WHK, Sumalee A, Li Q and Li ZC used an impact-area vulnerability analysis approach to identify critical links in large-scale and congested road networks [19]. Almotahari A and Yazici M A proposed a link criticality index (LCI) solved through an iterative process of user equilibrium traffic assignment formulation [20]. Tampère CMJ, Stada J, Immers B, Peetermans E and Organe K established criteria based on flow and capacity to select candidate links [21]. Then, incidents were simulated for these candidate links to identify the critical links. Knoop V L, Snelder M, van Zuylen H J and Hoogendoorn SP tested link-based strategies (e.g., a congestion indicator) for pre-selecting potential critical links [22]. They found that such strategies could not accurately characterize the impact of link interruption on the whole network and suggested that new, high-quality criteria for selecting candidate critical links should be found.

Betweenness is an important criterion for identifying critical components in complex networks, defined as the ratio of the shortest paths passing through the node or edge to all the shortest paths in the network [23–25], betweenness can avoid repeated iterations required by the traditional full-scan method. For this reason, we extend betweenness to establish a traffic flow betweenness index (*TFBI*) for identifying critical links in large-scale road networks. Gauthier P, Furno A and EI Faouzi N E deduced that a betweenness index could improve calculation efficiency, but accuracy could not be guaranteed [26]. It is expected that an approach for identifying critical links for large-scale road networks can not only improve computational efficiency but also be as accurate as possible. The traffic flow on a link implies drivers’ actual route choice behaviours and indicates the number of vehicles affected by the link interruption. In addition, if some endpoint of the interrupted link is an OD node, the affected travel demand will be rerouted. In view of the above, in addition to betweenness, the proposed *TFBI* also accounts for link traffic flow and rerouted travel demand. Based on the *TFBI*, an approach is proposed to identify critical links. The procedure of the *TFBI*-based approach consists of the following steps. First, the weight coefficient in the *TFBI* formula is calibrated according to a sample road network that is purposefully selected. Then, the links in the whole road network are ranked according to their *TFBI* values to select the candidate critical links. Finally, using the traditional full-scan method, the candidate critical links are ranked in descending order according to their *NRI* values [10] to identify the preferred number of critical links. Compared with existing related research [17–26], the main contributions of this study are as follows.

1. To reduce the computational burden, a novel *TFBI*-based approach is proposed to identify critical links in large-scale road networks for the first time. Its validity and computational efficiency have been tested on a real large-scale road network. The proposed approach incurs a lower computational burden than the traditional full-scan method.
2. Rather than the shortest distance path betweenness that is widely used in complex networks, the shortest travel-time path betweenness is adopted to establish the *TFBI*. This reflects the fact that drivers tend to choose the shortest travel-time path in an urban road network.
3. A link is assumed to be interrupted when the traditional full-scan method is used to evaluate network efficiency reduction. This might result in rerouted travel demand, which is not often mentioned in the literature. The proposed *TFBI* considers rerouted travel demand and its assignment.
4. The identification result of the systematic index tends to be more accurate than that of the link-based index [13]. The *TFBI* is a link-based index used to pre-select candidate critical links. Unlike the previous link-based index [21, 22], the systematic index *NRI* is used to calibrate the coefficient of the *TFBI*.

## Traffic flow betweenness index (*TFBI*)

Generally, betweenness roughly indicates the consequences of link closure [27]. In addition, owing to the great reduction in the computational burden when using betweenness to identify critical links, we establish the *TFBI* based on betweenness. In addition to betweenness, traffic flow should also be considered. Traffic flow is a very important factor that represents traffic distribution and density in the network. To a certain extent, betweenness can provide a rough representation of patterns of traffic flow or traffic density [28, 29], but it is not always accurate [26, 30–33]. For example, if the traffic flow going through a link with a larger value of betweenness is less, then fewer affected vehicles will have to alter their routes, even when this link is interrupted. Accordingly, links with larger values of betweenness do not always have as much influence on the efficiency of the entire road network. The traffic flow of a link implies the number of drivers that have chosen this link and how many vehicles are affected when this link is interrupted. If some endpoint of the interrupted link is an OD node, the travel demand on this OD node will be affected and have to be rerouted to an alternative OD node. Therefore, it is reasonable that betweenness, link traffic flow, and rerouted travel demand (which we refer to as the “endpoint OD demand”) are considered to establish a new link-based indicator, the *TFBI*, to indicate the criticality of the link. The *TFBI* comprises two components: traffic flow betweenness (*TFB*), which is a function of betweenness, link traffic flow, and total OD demand and endpoint OD demand.

Based on link betweenness, *TFB* is defined as a function of link traffic flow and total OD demand, as shown in formula (1):

$$TFB_{a}=\frac{\sum_{i,j\in V}n_{ij}(a)}{N}\cdot \frac{q_{a}}{D}$$ (1)

where *n*_*ij*_(*a*) is the shortest travel-time path through link *a* between origin node *i* and destination node *j*. $\sum_{i,j\in V}n_{ij}(a)$ is the total number of shortest paths between all the OD pairs that go through link *a*. *V* is the set of OD nodes, and *N* is the total number of shortest paths in the whole road network. *D* is the total OD travel demand of the whole road network. *q*_*a*_ is the traffic flow of link *a*.

$\frac{\sum_{i,j\in V}n_{ij}(a)}{N}$ is the shortest travel-time path betweenness of link *a*. It reflects how many shortest paths will be altered when link *a* is disrupted. The shortest travel-time path betweenness implies the link criticality under the assumption that drivers prefer the shortest routes. *q*_*a*_ is the real (surveyed) traffic flow of link *a*, which represents the number of drivers that have chosen link *a*. *q*_*a*_ implies drivers’ actual route choice behaviours. *q*_*a*_ represents the loaded OD demand between every OD pair that goes through link *a*, and *q*_*a*_*/D* reflects how large a share of OD demand is affected when link *a* is disrupted.

If some endpoint of the interrupted link is an OD node, the affected travel demand will be routed to other OD nodes. The endpoint OD demand is loaded at its closest OD pair. We denote by *s* and *t* the endpoints of link *a*. If either *s* or *t* is an OD node, the endpoint demand *d*_*st*_, regardless of the traffic generation or traffic attraction, will be rerouted to the node nearest to the centroid of the same traffic zone in distance. This node will be a new OD node or an OD node in an adjacent traffic zone. When both *s* and *t* are OD nodes, the rerouted travel demand *d*_*st*_ equals the sum of the travel demand of *s* and the travel demand of *t*.

Endpoint OD demand will be redistributed, thereby altering the entire network’s efficiency. Accordingly, endpoint OD demand and *TFB* are considered together to calculate the *TFBI*, as shown in formula (2). The value of *TFBI*_*a*_ reflects the criticality of link *a*: the larger the value is, the more critical link *a* is.

$$TFBI_{a}={\left[{\left(TFB_{a}\right)}_{nor}\right]}^{r}\cdot {\left[{\left(d_{st}\right)}_{nor}\right]}^{1-r}$$ (2)

where *d*_*st*_ is the endpoint OD demand and thus the affected demand caused by the interruption of link *a*. If neither *s* nor *t* is an OD node, *d*_*st*_ = 1, and no rerouted travel demand results from the link disruption. Specifically, *TFBI*_*a*_ is related only to *TFB*_*a*_. (*TFB*_*a*_)_*nor*_ and (*d*_*st*_)_*nor*_ are the normalized values of *TFB*_*a*_ and *d*_*st*_, respectively, and *r* is the coefficient used to allocate weights between (*TFB*_*a*_)_*nor*_ and (*d*_*st*_)_*nor*_.

The value of *r* is between 0 and 1. The higher *r* is, the more important (*TFB*_*a*_)_*nor*_ is in the *TFBI*_*a*_ function. That is, the rank orders based on the *TFBI* will be more consistent with the *TFB*. *r* = 0 and *r* = 1 indicate that the links are ranked by endpoint OD demand and *TFB*, respectively. The value of *r* is determined according to systematic indicators. The calibration of the weight coefficient *r* is described in the section “Procedure for the *TFBI*-based approach”.

## *TFBI*-based approach for identifying critical links

To improve computational efficiency, we propose a *TFBI*-based approach for identifying critical links in large-scale road networks. In this approach, first, a systematic indicator, such as *NRI*, is used to calibrate the weight coefficient *r* in the *TFBI*. Then the *TFBI* values of all the links in the road network are calculated. All links are ranked in descending order according to their *TFBI* values. Based on the order of the links, a certain number of links is selected from top to bottom as the set of candidate critical links. Finally, the traditional algorithm is used to calculate the value of the systematic index (*NRI*) for candidate critical links. Thus, the critical links are determined according to the given number of critical links. The basic datasets used in this study are in S1 File. The primary data were obtained from Changchun traffic big data platform of Changchun Municipal Engineering Design and Research Institute, which is a partner of the project supporting our study. We extracted the basic data used in our study, such as link properties, link capacity, link traffic flow, and link free-flow travel time, from the primary data.

### Network robustness index (*NRI*)

This article adopts the *NRI* as a criterion for calibrating the value of *r* in the *TFBI* and testing the *TFBI*’s efficiency. Other indicators can be adopted according to the actual situation of the city in which the approach is applied. The *NRI* and its algorithm are described as follows.

Scott et al. proposed the *NRI* for identifying critical links with regard to changes in the whole-system travel time (or the generalized cost) resulting from each link closure using the traditional full-scan method [10]. The *NRI* reflects variations in network efficiency and considers other factors, such as network topology and traffic flow. The *NRI* is calculated, as shown in formula (3). The larger the *NRI*_*a*_ value is, the more critical link *a* is.

$$NRI=\sum_{a}t_{a}^{\prime }(x_{a})x_{a}^{\prime }\delta_{a}-\sum_{a}t_{a}(x_{a})x_{a}$$ (3)

where *x*_*a*_ and *t*_*a*_(*x*_*a*_) are the traffic flow and travel time of link *a*, respectively, when no link is interrupted. *x*_*a*_^′^ and *t*_*a*_^′^(*x*_*a*_) are the traffic flow and travel time of link *a*, respectively, when a link is interrupted. *t*_*a*_(*x*_*a*_) and *t*_*a*_^′^(*x*_*a*_) represent the relationship between the traffic flow and travel time for link *a*. If link *a* is removed, *δ*_*a*_ = 0; otherwise, *δ*_*a*_ = 1.

In our study, the travel time of link *a* is calculated based on the widely used *BPR* (the Bureau of Public Roads) function, which was proposed by U.S. Bureau of Public Roads, as shown in formula (4).

$$t_{a}(x_{a})=t_{0a}(1+\alpha {\left(x_{a}/c_{a}\right)}^{\beta })$$ (4)

where *t*_*0a*_ is the free-flow travel time on link *a*, *c*_*a*_ is the capacity of link *a*, and *α* and *β* are the parameters.

*α* and *β* are set by fitting the *BPR* function to actual survey data. First, formula (4) is transformed into a linear equation to be fitted (such as *y = a + bx*). Then, the surveyed data, *x*_*a*_, *c*_*a*_, *t*_*0a*_, and *t*_*a*_(*x*_*a*_), are input into formula (5), and *α* and *β* are obtained by linear fitting:

$$\text{ln}(t_{a}(x_{a})/t_{0a}-1)=\text{ln}\alpha +\beta \text{ln}(x_{a}/C_{a})$$ (5)

To calculate the *NRI*, each link is removed in sequence. Once a link has been removed, the traffic flow is redistributed in the changed network using the user equilibrium assignment (UE) model [34]. The formulas are as follows.

$$\text{min}f_{2}=\sum_{a}\int_{0}^{x_{a}}t_{a}(\omega )d_{\omega }$$ (6)

s. t.

$$\sum_{k}f_{k}^{rs}=q_{rs},\forall \;r,s$$ (7)

$$f_{k}^{rs}\geq 0,\forall \;r,s$$ (8)

$$x_{a}=\sum_{r,s}\sum_{k}f_{k}^{rs}\delta_{a,k}^{rs},\forall \;a$$ (9)

where *f*_*k*_^*rs*^ is the traffic flow of the *k*-th path between OD nodes *r* and *s*, and *q*_*rs*_ is the OD demand between *r* and *s*. $\delta_{a,k}^{rs}$ represents 0–1 variables. $\delta_{a,k}^{rs}=1$ indicates link *a* on path *k*; otherwise, $\delta_{a,k}^{rs}=0$.

The steps to solve the UE model are as follows.

Step 1: Set *k* = 1 and maximum number of iterations *n*.
Step 2: Obtain the initial link traffic flow *x*_*a*_^0^ by using the all-in-all-nothing assignment method according to the link travel time when there is no traffic flow in the road network.
Step 3: Update *t*_*a*_(*x*_*a*_) according to the *BRP* function.
Step 4: Obtain additional traffic flow *z*_*a*_^*k*^ by the all-or-nothing assignment method.
Step 5: Determine the iteration step size λ. *λ* = 1/*k*.
Step 6: Update the link traffic flow according to the formula $x_{a}^{k+1}=(1-\lambda )x_{a}^{k}+\lambda z_{a}^{k}$.
Step 7: End the calculation if *k* > *n*; otherwise, returns to Step 3.

### Procedure for the *TFBI*-based approach

The procedure for the *TFBI*-based approach consists of three main steps:

Step 1: Calibrate the value of *r* in the *TFBI* based on the sample road network.
  1. Select a sample road network from the whole road network. The criteria for the sampling network are introduced in the next section. Then, obtain the OD travel demand matrix of the sample road network. The travel demand *d*_*ij*_ between node *i* and node *j* is obtained by survey-based methods or estimated by link traffic flow [35]. Obtain the corresponding OD demand matrix [*d*_*ij*_] and the total amount of demand *D*.
  2. Determine the shortest travel-time path between every OD pair in the road network. The shortest path is obtained using the depth-first search algorithm according to the adjacency matrix. This adjacency matrix is constructed of the travel time between adjacent nodes. If there are multiple shortest paths between an OD pair, one of these shortest paths is chosen at random. Thus, ∑*n*_*ij*_ (*a*) and *N* are obtained.
  3. Input the data obtained from the foregoing steps into formula (1) to calculate *TFB*_*a*_. Obtain *q*_*a*_ through actual investigation.
  4. Determine coefficient *r* by maximizing Spearman’s rank correlation coefficient *ρ* between the *TFBI* rank and the *NRI* rank. Calculate *ρ*, as shown in formula (10) [36]. Let $R_{i}^{FBI}$ be the rank of the measurement of *TFBI*_*a*_ taken on the *i* th individual, and let $R_{i}^{NRI}$ be defined in the same way using the *NRI* as the measurement. *n* is the total number of links in the road network. The ranking based on the *TFBI* is expected to be very highly and positively correlated with the ranking based on the *NRI*. If the value of *ρ* is approximately 1, the value of coefficient *r* is optimal. If *ρ* = 1, *r* is ideal. However, *ρ* depends on the variance in $R_{i}^{FBI}$ and $R_{i}^{NRI}$; thus, there can be a case in which *ρ* cannot equal 1. The optimal value of *r* is determined by minimizing 1-*ρ*.

$$\rho =1-\frac{6\sum {\left(R_{i}^{FBI}-R_{i}^{NRI}\right)}_{i}{}^{2}}{n(n^{2}-1)}$$ (10)

The problem of minimizing 1-*ρ* is formulated as an optimization problem as follows:

$$Min\;Y(r)=\frac{6\sum {\left(R_{i}^{FBI}-R_{i}^{NRI}\right)}_{i}{}^{2}}{n(n^{2}-1)}$$ (11)

s.t. 0≤*r*≤1

First, when *r* equals 0 or 1, the value of *Y*(*r*) is not at its minimum value. Then, the gradient flow method is adopted to solve the optimization model.

Let

$${\text{Y}}_{\varepsilon }(r)=\frac{Y(r+\varepsilon )-Y(r-\varepsilon )}{2\varepsilon },\;r\in [\varepsilon ,1-\varepsilon ]$$ (12)

Set *ε* = 0

Let

$$y=sign(x)=\left\{ \begin{array}{cc} 1 & x>0\\ 0 & x=0\\ -1 & x<0 \end{array}\right.$$ (13)

**Fig 1:**
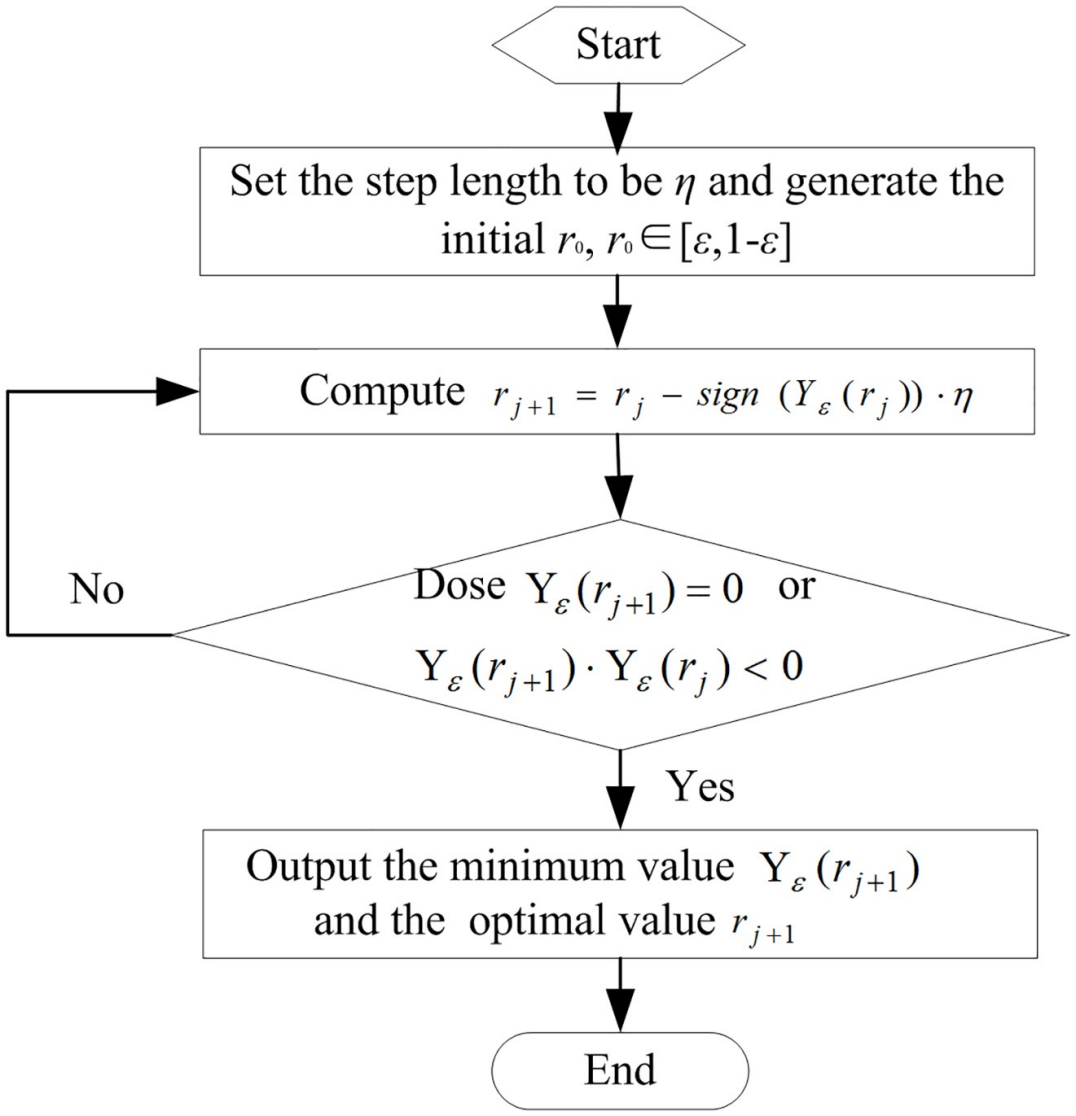
Flowchart of the gradient flow method.

The flowchart of the gradient flow method is shown in Fig 1.

Step 2: Apply the *TFBI* to the whole road network to select candidate critical links.
  1. Based on the value of *r*, put *TFB*_a_ and *d*_*st*_ into formula (2) to calculate the value of the *TFBI*_*a*_ for each link in the whole road network. The method for obtaining the *TFBI* variables, such as *n*_*ij*_, *N*, *D*, and *q*_*a*_, is described in Step 1 parts (1) and (2).
  2. Rank all links in the road network in descending order according to their *TFBI*_*a*_ values. Then, select a certain number of links as the set of candidate critical links.
Step 3: Compute the *NRI*_*a*_ for all the candidate critical links and rank them in descending order according to their *NRI*_*a*_ values. Then, according to the given number of critical links, determine the real critical links from the candidate critical links.

**Fig 2:**
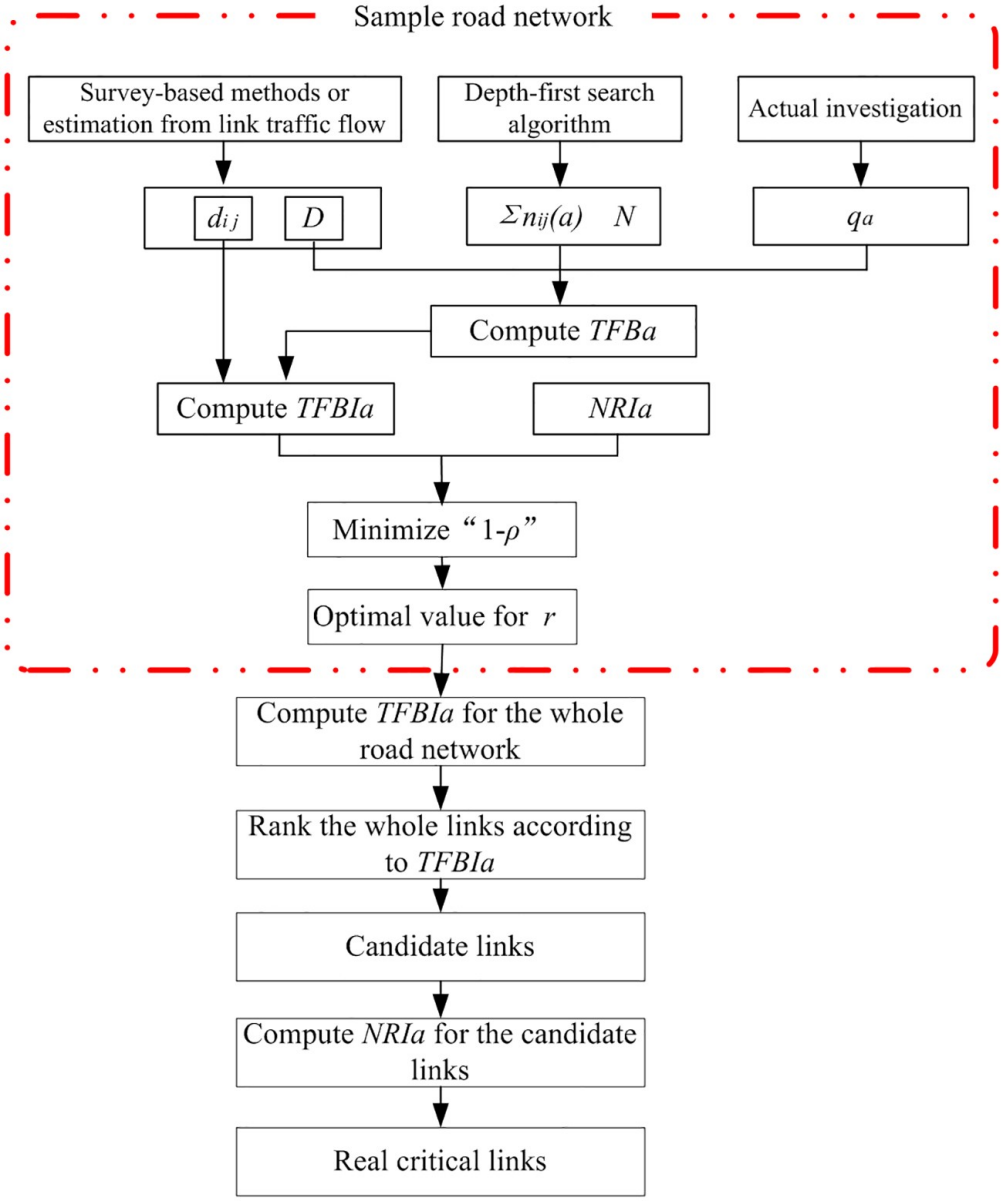
Procedure for the *TFBI*-based approach.

Based on the above procedure, the algorithm of the *TFBI*-based approach is shown in Fig 2. The data for the calibration of *r* in the pane with the dashed line are calculated based on the sample road network.

### Criteria for the sample road network

The weight coefficient *r* should make the *TFBI* ranking consistent with the *NRI* ranking. The value of *r* in the *TFBI* is calibrated based on the sample road network. The criteria for potential sample road networks are as follows and sensitivity analysis tests were carried out. The rationality of the criteria was tested based on the sample road network which is introduced in detail in the section “Criteria for sample road network”.

1. The partial network located in the central business district (CBD) is suggested as the sample road network. With regard to the selection of the location of the sample road network, the OD node should be in the selected area. Namely, the endpoint OD demand *d*_*st*_ should be in the sample road network.
  The *TFBI* is calculated according to formula (2), which comprises three main factors: the link betweenness, the link traffic flow, and the endpoint OD demand *d*_*s*_. If a sample road network is selected from an uncongested area, there may be no affected demand *d*_*st*_. Enlarging the scope of the sample size to obtain *d*_*st*_ would obviously not be in line with this paper’s purpose of reducing the computational burden. The CBD is more likely to contain *d*_*st*_ and have a large value of link traffic flow. Thus, the partial network located in the CBD is suggested as the sample road network.
2. The sample road network should include all road types in the given road network, such as trunk roads, secondary trunk roads, and branches, to make the sample road network more representative of the whole road network.
3. On the basis of meeting the above conditions (1) and (2), 10‰ is the suggested minimum percentage of links from the total network in the sample road network.

In general, the larger the sample road network is, the higher the accuracy of the proposed *TFBI* and the longer the computation time. Thus, it is necessary to consider the trade-off between accuracy and computational performance in choosing the appropriate size for the sample road network.

**Fig 3:**
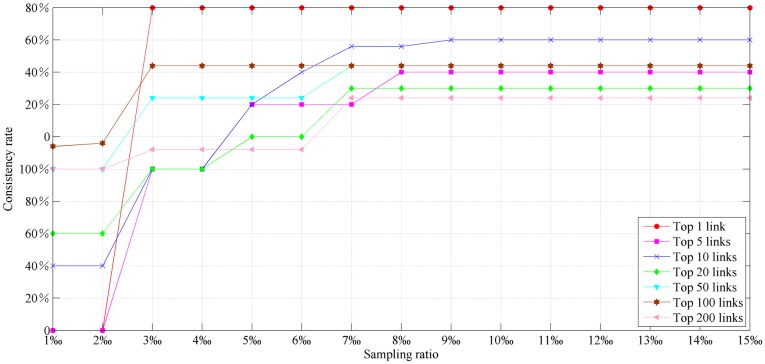
Consistency rates between the *TFBI* and *NRI* with different sampling ratios.

Under the condition of meeting the above criteria (1) and (2), it is suggested that the number of links in the sample road network should be as low as possible to reduce the calculation burden. The values of sampling ratio (the ratio of the number of links in the sample road network to the number of links in the whole network) were taken to be 1‰, 3‰, 5‰, 7‰, 10‰, and 15‰. Compared to the *NRI*, the consistency rates of the top 1, 5, 10, 20, 50, and 100 links based on the *TFBI* with different sampling ratios are shown in Fig 3. The consistency rate is used to measure the accuracy of the *TFBI* and refers to how many critical links identified by the *TFBI* are in the range of critical links identified by the *NRI*. For example, of the top 5 links identified by the *TFBI*, four are in the range of the top 5 links identified by the *NRI*, and the consistency rate is 80%. From Fig 3, the consistency rate increases with an increase in the sampling ratio. When the sampling ratio is less than 5‰, the consistency rate grows faster as the sampling ratio increases. When the sampling ratio is greater than 5‰, the rate of growth is slow. When the sampling ratio is 10‰, the rate of growth is stable. Based on the above analysis, 10‰ is the suggested minimum sampling ratio relative to the total network.

## Case study

The *TFBI*-based approach proposed in this paper is implemented using the road network of Changchun, China, to validate its feasibility. The case study is organized as follows: First, the travel data for the Changchun road network are obtained. Second, the traditional full-scan method based on the *NRI* is used to rank all links of the Changchun road network. Then, the *TFBI*-based approach is used to identify critical links, and the computational performance of the *TFBI*-based approach and *NRI* are compared.

### Changchun road network and travel data

**Fig 4:**
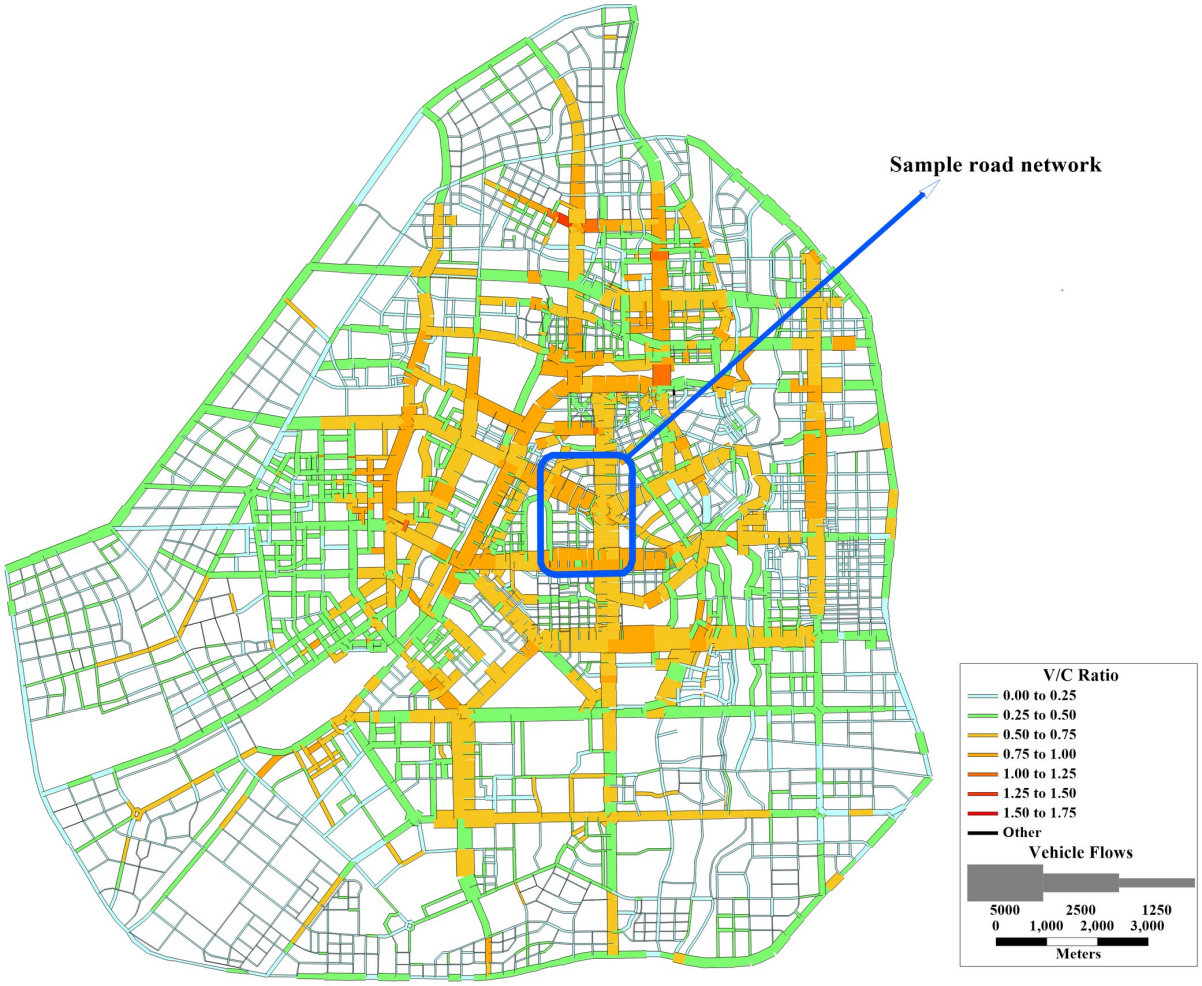
The V/C ratio and traffic flow of all links in the Changchun road network.

Changchun is the capital of Jilin Province, which is located in the middle of northeast China. Changchun’s road network consists of 3,207 links surrounded by ring roads, 1,122 intersections, and 201 zones. To simplify the calculation, the intersections are assumed to be nodes. The intersection closest to the centroid of the traffic zone (in distance) is taken as the OD node. The link traffic flow in the peak evening hours (17:00–18:00) is selected for estimating the OD demand matrix. Accordingly, among 18,090 OD pairs, a total of 68,830 trips in passenger car units were observed. In this case study, the OD demand matrix is estimated according to the surveyed link traffic flow [35]. The ratio of traffic flow to capacity (V/C) and traffic flow are shown in Fig 4, which is outputted by TransCAD.

The travel time is calculated by the widely used *BPR* function. Based on actual survey data from the sampling network, the parameters in the *BPR* function are set to *α* = 0.28 and *β* = 2.35 by fitting the *BPR* function according to formula (5). To simplify other influencing factors, our hypotheses are as follows. (1) The evacuation of vehicles on an interrupted link is not considered. (2) If the endpoint of an interrupted link is an OD node, the unsatisfied OD demand is rerouted to the nearest OD node, and the total travel demand of the network is fixed.

### Identifying critical links with *NRI*

**Fig 5:**
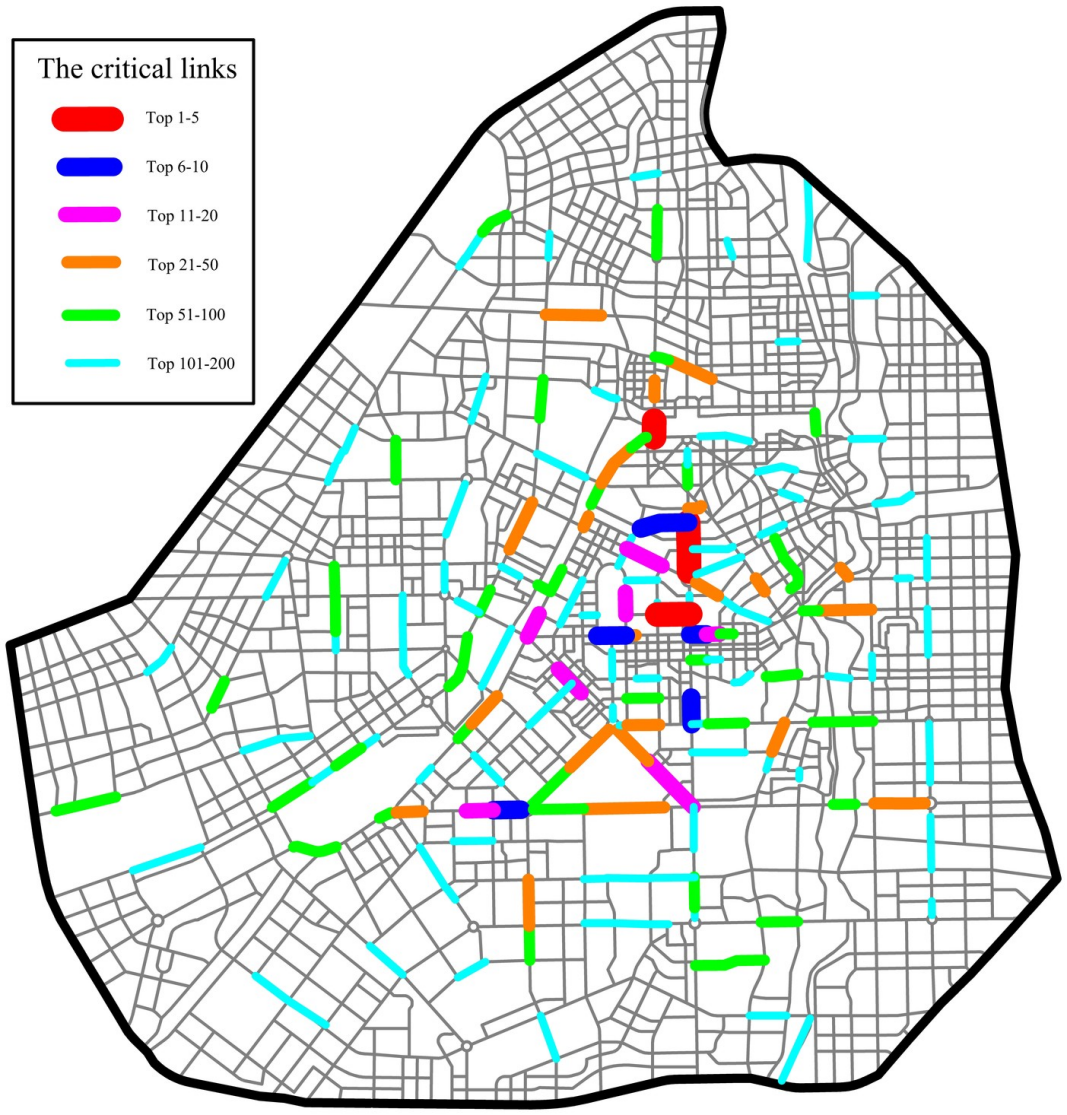
Critical links based on the *NRI* in the Changchun road network.

The 3,207 links in the Changchun road network are ranked in descending order according to their *NRI* values. The calculation of the *NRI* value of each link is introduced in detail in the section “Network robustness index (*NRI*)”. As in the *TFBI*-based approach, if some endpoint of the interrupted link is an OD node, the rerouted travel demand is loaded at its closest OD pair. The top 250 links are identified as critical links, as shown in Fig 5. Most of the critical links are located in the central area of Changchun City. Not all of the crowded links in the central area are critical links, even though they have a high travel demand because some have alternative links owing to the high density of the network.

**Fig 6:**
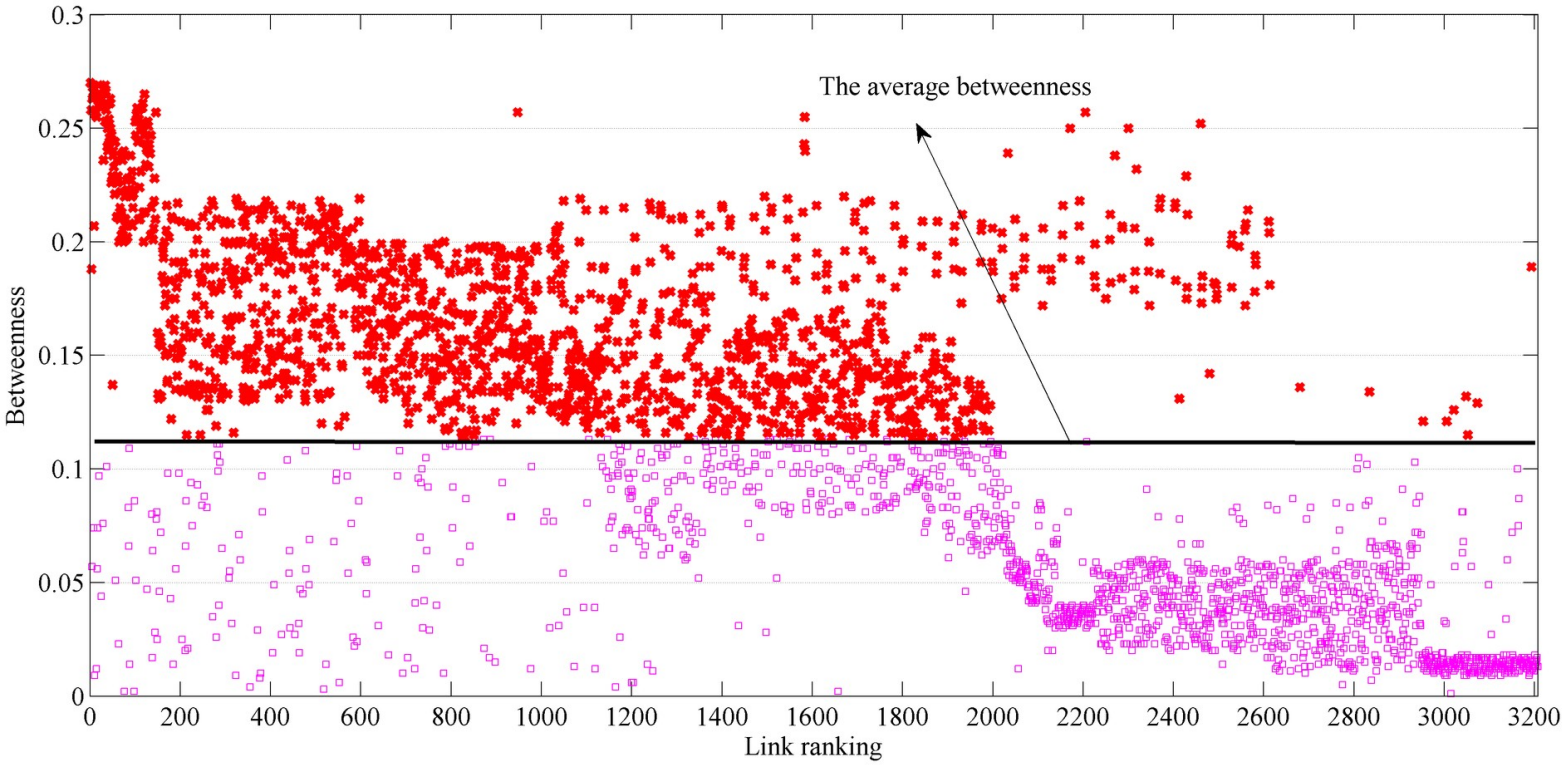
Betweenness of each link.

**Fig 7:**
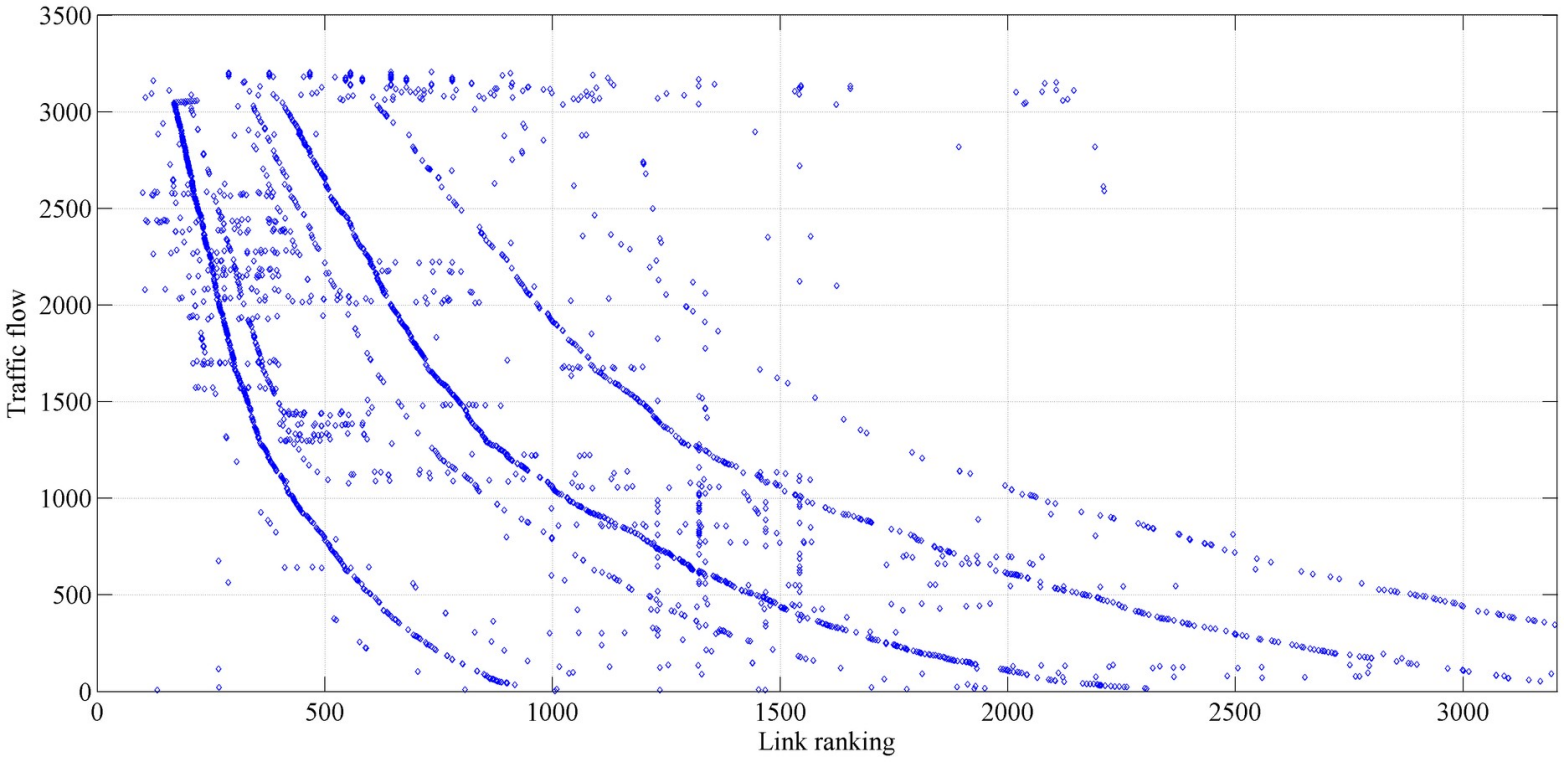
Traffic flow of each link.

The betweenness and traffic flow characteristics of the critical links are shown in Figs 6 and 7. The *x*-axis represents the sequence of links in descending order, ranked by the decrease in network efficiency when the corresponding link is removed. The reduction in network efficiency is calculated with respect to the *NRI*. Most of these critical links (identified by the *NRI*) have high betweenness and heavy traffic flow. Additionally, the link ranks according to betweenness and traffic flow are inconsistent. In this study, link betweenness and traffic flow are used to establish *TFB*.

### Identifying critical links with the *TFBI*-based approach

In practice, due to budget limitations, decision makers or transport planners only want to identify a small number of the most critical links to better protect or link capacity to enhance to resist and respond to emergencies. The traditional algorithm is that in which the deduction of the road network efficiency is assessed due to each link’s failure. For large-scale road networks, computationally intensive work is needed to determine the small number of critical links. The proposed *TFBI*-based approach can solve this practical problem because it avoids repeated traffic assignments under all scenarios of link disruption. In this section, the applicability of the *TFBI*-based approach is demonstrated in the Changchun road network. When the proposed approach is applied to the city road network, the optimal value of coefficient *r* in the *TFBI* is determined with reference to the sample network data first. Then, all links in the road network are ranked according to their *TFBI* values to select candidate critical links. These candidate critical links are then ranked according to their *NRI* values to determine a given number of real critical links that decision makers or transport planners require.

**Fig 8:**
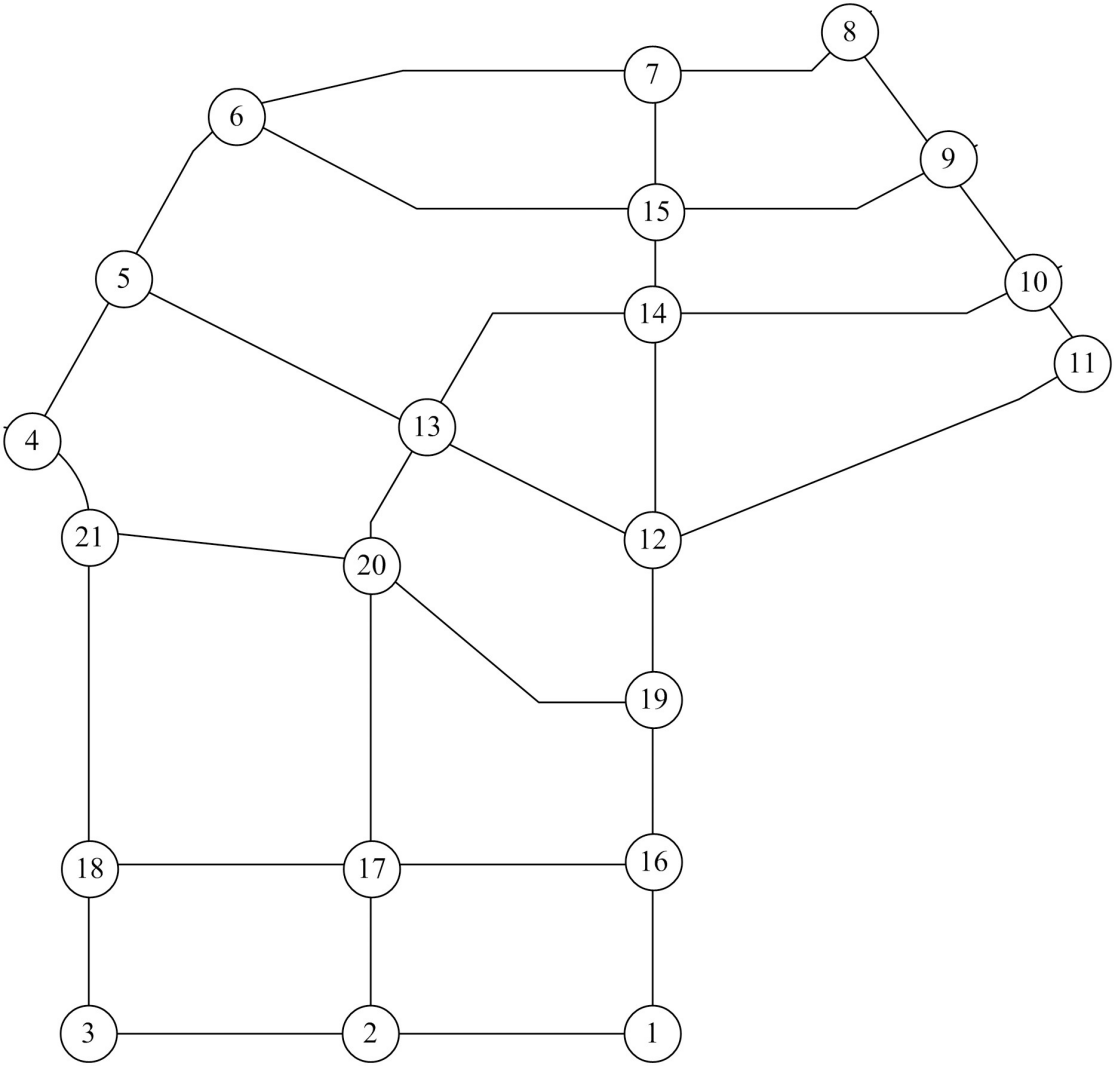
Sample road network.

**Fig 9:**
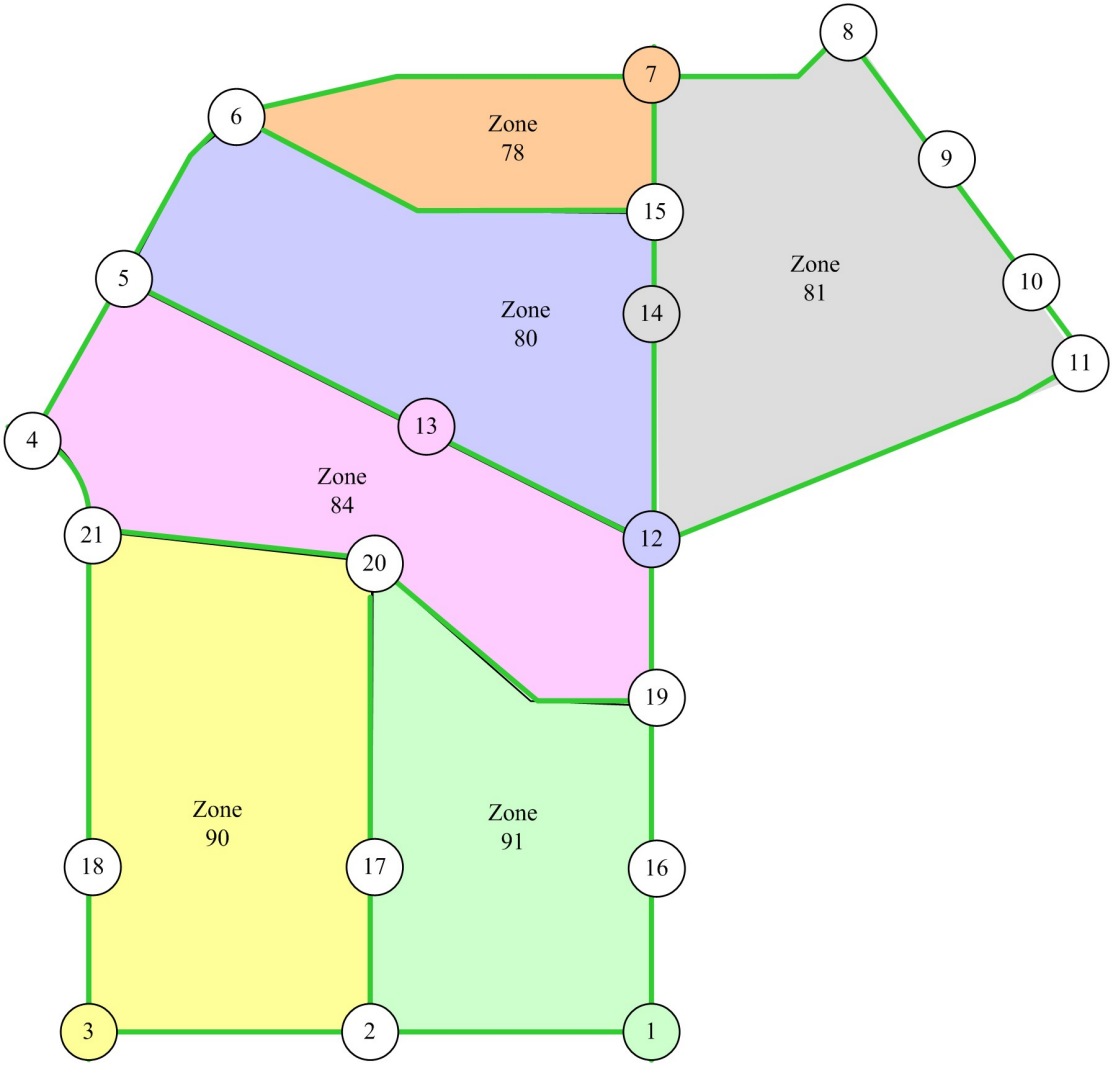
Traffic zones of the sample road network.

The optimal value of coefficient *r* in formula (2) should be determined before the *TFBI* is applied in the road network. According to the criteria for the sample road network, a portion of the Changchun City road network (see Fig 4) is selected to serve as the sample road network for calibrating coefficient *r*. There are 21 nodes, 32 links, and 6 zones in the sample road network, as shown in Fig 8. The centroid of a zone is converted into its nearest node in distance. This node is regarded as an OD node, where traffic demand is generated and attracted. Nodes 1, 3, 7, 12, 13, and 14 are OD nodes, as shown in Fig 9. Fifteen OD pairs observed a total of 13,766 trips in passenger car units.

Coefficient *r* determines the distribution weight between the *TFB* and endpoint OD demand. In statistical analysis, we commonly explore and summarize the strength of the association between two measured traits [37]. Using sampling links, the optimal coefficient *r* is determined through an analysis of Spearman’s rank correlation between the *TFBI* and *NRI* rank orders. The detailed solution process of the optimal coefficient *r* can be found in the section “Procedure for *TFBI*-based approach”. The results of the *TFBI* fit the results of the *NRI* best (*ρ* = 1) when *r* = 0.55. The comparison of the normalized *TFBI* and *NRI* values in Table 1 clearly indicates that the different calculation processes of the two indexes lead to different criticality values. From the results of Table 1, betweenness, *TFB* or endpoint OD demand cannot exactly indicate the criticality of the link. By comparison, the identification results of the critical links with the *TFBI*-based approach tend to be more accurate because the coefficient *r* in the *TFBI* is calibrated by the systematic index *NRI*.

**Table 1:** Results for *TFBI* (*r* = 0.55) compared with those for *NRI*.

*NRI*			*TFBI*(*r* = 0.55)					
Link	Normalized value	Rank	Link	Betweenness	(*TFB*)_*nor*_	(d_st_)_*nor*_	Normalized value	Rank
**1–2**	0.1116	1	**1–2**	0.1714	0.0771	0.3274	0.2014	1
**12–13**	0.0576	2	**12–13**	0.1238	0.0928	0.1287	0.1465	2
**13–14**	0.0576	3	**13–14**	0.0571	0.0485	0.1017	0.0922	3
**7–8**	0.0571	4	**7–8**	0.1333	0.1062	0.0214	0.0704	4
**1–16**	0.0433	5	**1–16**	0.1667	0.1050	0.0214	0.0700	5
**5–13**	0.0378	6	**5–13**	0.1238	0.0834	0.0121	0.0477	6
**7–15**	0.0356	7	**7–15**	0.1429	0.0088	0.1063	0.0368	7
**8–9**	0.0314	8	**12–14**	0.0762	0.0944	0.0055	0.0358	8
**12–14**	0.0296	9	**8–9**	0.1905	0.0335	0.0195	0.0358	9
**14–15**	0.0296	10	**14–15**	0.2571	0.0490	0.0084	0.0302	10
**2–3**	0.0290	11	**2–3**	0.1333	0.0377	0.0084	0.0261	11
**13–20**	0.0290	12	**13–20**	0.0300	0.0318	0.0097	0.0254	12
**2–17**	0.0274	13	**2–17**	0.0200	0.0311	0.0084	0.0235	13
**10–14**	0.0265	14	**10–14**	0.0190	0.0276	0.0084	0.0220	14
**5–6**	0.0263	15	**5–6**	0.1714	0.0261	0.0063	0.0188	15
**4–5**	0.0261	16	**4–5**	0.0476	0.0411	0.0032	0.0178	16
**3–18**	0.0257	17	**3–18**	0.1333	0.0271	0.0032	0.0141	17
**6–15**	0.0255	18	**6–15**	0.1524	0.0036	0.0203	0.0107	18
**9–10**	0.0253	19	**9–10**	0.0400	0.0042	0.0114	0.0090	19
**9–15**	0.0248	20	**11–12**	0.0571	0.0116	0.0032	0.0089	20
**11–12**	0.0248	21	**9–15**	0.0952	0.0099	0.0032	0.0081	21
**17–20**	0.0246	22	**17–20**	0.0381	0.0117	0.0023	0.0077	22
**10–11**	0.0245	23	**10–11**	0.1905	0.0005	0.0971	0.0073	23
**6–7**	0.0244	24	**6–7**	0.0857	0.0015	0.0245	0.0072	24
**12–19**	0.0231	25	**18–21**	0.2667	0.0084	0.0028	0.0070	25
**4–21**	0.0230	26	**4–21**	0.0400	0.0150	0.0010	0.0060	26
**18–21**	0.0220	27	**12–19**	0.0600	0.0053	0.0017	0.0043	27
**16–17**	0.0171	28	**16–17**	0.0200	0.0029	0.0010	0.0024	28
**16–19**	0.0169	29	**20–21**	0.0100	0.0018	0.0015	0.0023	29
**17–18**	0.0151	30	**16–19**	0.1467	0.0002	0.0171	0.0020	30
**20–21**	0.0140	31	**17–18**	0.0400	0.0002	0.0127	0.0018	31
**19–20**	0.0139	32	**19–20**	0.0100	0.0023	0.0001	0.0008	32

**Fig 10:**
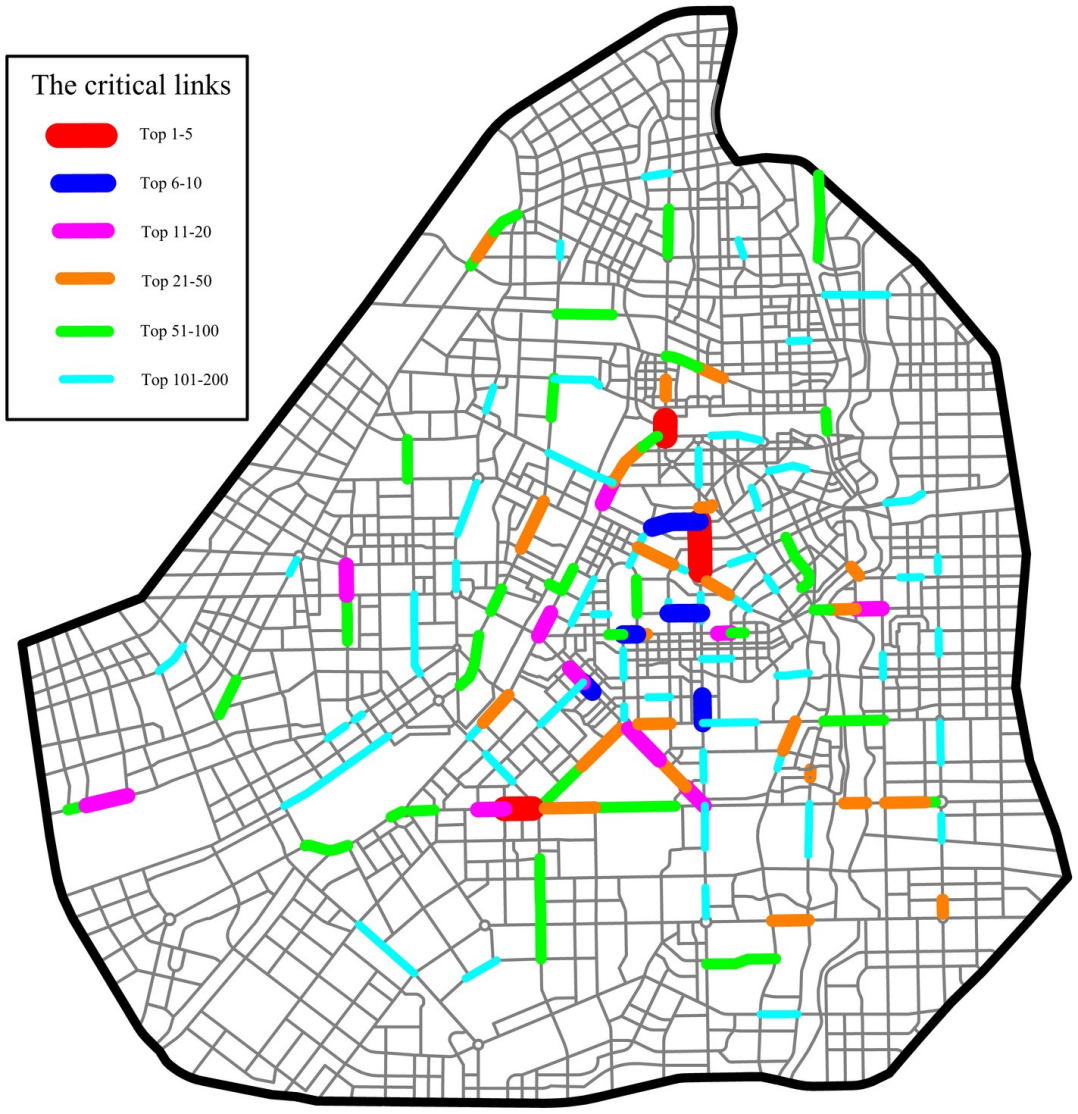
Critical links based on the *TFBI* in the Changchun road network.

The 3,207 links in the Changchun road network are ranked in descending order according to their *TFBI* values. *TFBI*_*a*_ is calculated by formula (2). The top 250 critical links are identified, as shown in Fig 10. Most of the critical links are located in the CBD, where rerouted demand (endpoint OD demand) is heavy as a result of the interruption of these critical links. Other critical links have large betweenness values and fewer alternative links.

When *r* takes different values, the consistency rates of the critical links and Spearman’s rank correlation coefficient *ρ* between the *TFBI* and *NRI* are as presented in Table 2. We can see that the value of *r* affects the accuracy of the *TFBI*. When *r* = 0.55, the consistency rates of the critical links and Spearman’s rank correlation coefficient *ρ* between the *TFBI* and *NRI* are largest. *ρ* is computed across all the links of the whole road network.

**Table 2:** Consistency rates of critical links and Spearman’s rank correlation coefficient *ρ* between the *TFBI* and *NRI*.

*r*	Consistency rates of critical links (%)							*ρ*
	Top 1 link	Top 5 links	Top 10 links	Top 20 links	Top 50 links	Top 100 links	Top 200 links	
**0**	0%	0%	20%	30%	50%	57%	50%	0.60
**0.1**	0%	0%	20%	30%	50%	58%	50%	0.60
**0.2**	0%	0%	30%	50%	60%	58%	50%	0.68
**0.3**	100%	50%	50%	50%	70%	72%	60%	0.70
**0.4**	100%	50%	70%	70%	80%	82%	70%	0.73
**0.5**	100%	80%	88%	75%	82%	82%	72%	0.75
**0.55**	100%	80%	90%	75%	82%	84%	72%	0.75
**0.6**	100%	80%	88%	75%	82%	82%	72%	0.75
**0.7**	100%	50%	70%	75%	80%	82%	70%	0.74
**0.8**	100%	50%	50%	70%	72%	82%	70%	0.74
**0.9**	0%	0%	50%	50%	72%	82%	60%	0.70
**1**	0%	0%	50%	50%	68%	71%	60%	0.70

**Fig 11:**
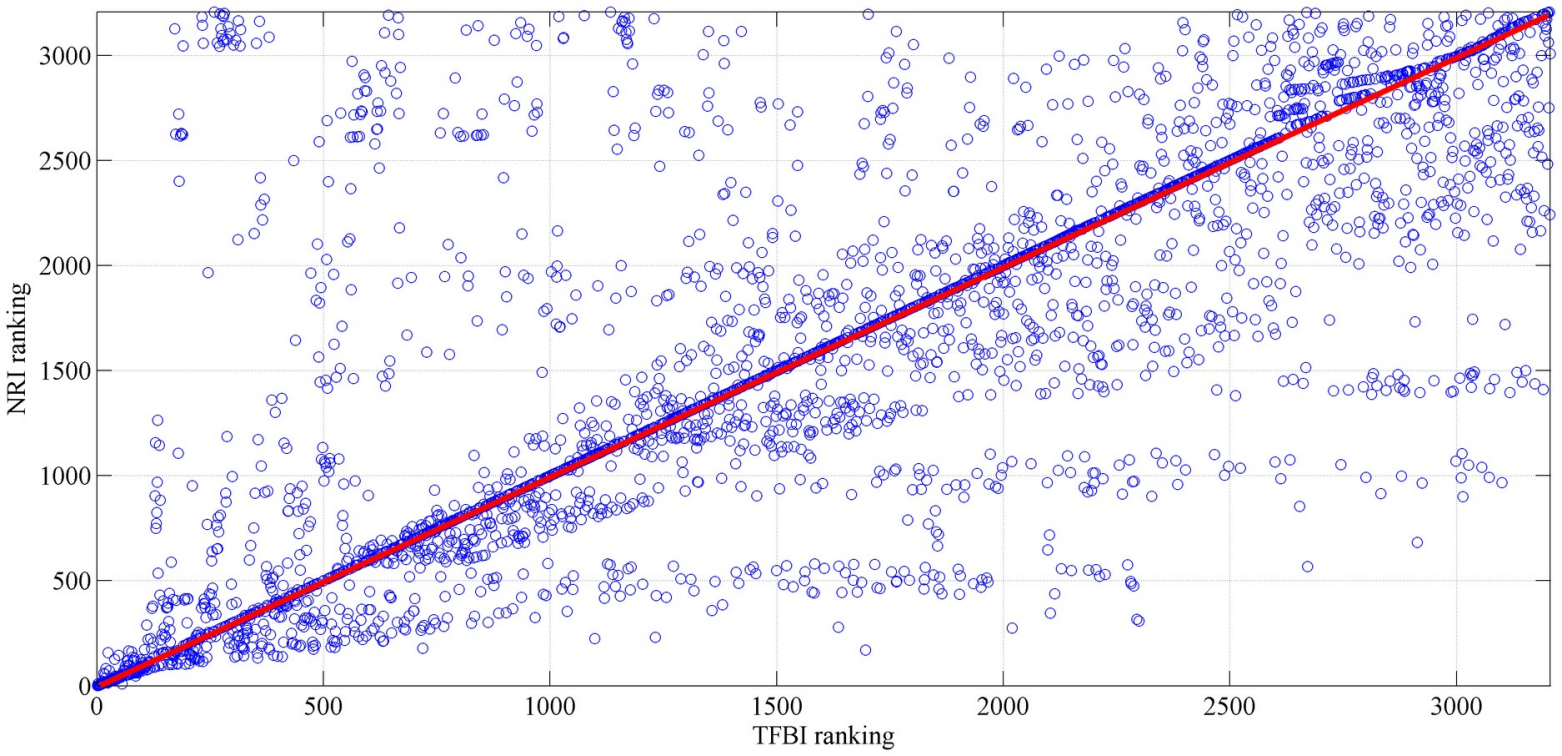
Correlation *ρ* between *NRI* and *TFBI* rankings when *r* = 0.55.

Fig 11 illustrates the correlation between the *NRI* and *TFBI* rankings when *r* = 0.55. The Spearman’s rank correlation coefficient *ρ* is 0.75 across all the links of the whole road network. A strong positive correlation is shown between the *TFBI* and *NRI* rankings. This result indicates that the critical links identified by the *TFBI* can be selected as the candidate critical links.

According to the *TFBI* values of links in descending order, a certain number of candidate critical links are pre-selected from top to bottom. The number of candidate critical links is multiple times the given number of real critical links. Based on the candidate critical links, the traditional full-scan algorithm is used to determine the given number of real critical links. Table 3 shows the consistency rates of the critical links between the *TFBI* (*r* = 0.55) and *NRI* when selecting different numbers of candidate critical links. The consistency rates increase with the number of candidate critical links. If the number of candidate critical links is not very large, compared with the given number of real critical links, some real critical links are omitted. Using *TFBI* to pre-select candidate critical links and then using the traditional full-scan method to identify the real critical links will improve the accuracy of the *TFBI*-based approach. We assume the 50 most critical links to be identified. Using the *TFBI* (*r* = 0.55) directly, the consistency rate with *NRI* is 82% (see Table 2). However, when using the *TFBI* to pre-select the top 150 or more candidate critical links, the consistency rate is 100%. The consistency rate varies with the number of candidate critical links. If we want to improve the identification accuracy, we can expand the number of candidate critical links.

**Table 3:** Consistency rates of critical links between *TFBI* (*r* = 0.55) and *NRI*.

Number of candidate critical links	Consistency rates of critical links (%)						
	Top 1 link	Top 5 links	Top 10 links	Top 20 links	Top 50 links	Top 100 links	Top 200 links
**5**	100%	80%	-	-	-	-	-
**10**	100%	100%	90%	-	-	-	-
**20**	100%	100%	90%	75	-	-	-
**50**	100%	100%	90%	90%	82%	-	-
**100**	100%	100%	100%	100%	98%	84%	-
**150**	100%	100%	100%	100%	100%	93%	-
**200**	100%	100%	100%	100%	100%	95%	72%
**250**	100%	100%	100%	100%	100%	95%	81%
**300**	100%	100%	100%	100%	100%	97%	82%
**350**	100%	100%	100%	100%	100%	98%	86%
**400**	100%	100%	100%	100%	100%	99%	89%
**450**	100%	100%	100%	100%	100%	99%	92%
**500**	100%	100%	100%	100%	100%	99%	93%
**600**	100%	100%	100%	100%	100%	100%	95%
**700**	100%	100%	100%	100%	100%	100%	96%
**750**	100%	100%	100%	100%	100%	100%	96%
**800**	100%	100%	100%	100%	100%	100%	97%
**900**	100%	100%	100%	100%	100%	100%	97%
**1000**	100%	100%	100%	100%	100%	100%	97%
**1200**	100%	100%	100%	100%	100%	100%	99%

The computational performance of the *TFBI*-based approach is examined in this case study, and the results are shown in Table 4. MATLAB R2014a software is used for simulation on a computer with a quad-core 2.20 GHz CPU and 4GB of RAM. The computational performance of the traditional full-scan approach (the number of candidate critical links is set to 3,207) is evaluated using the same computer equipment. The computational performance is mainly affected by the following aspects: the computation time required for calibrating the coefficient *r* based on the sample network (denoted as *t*_*1*_), the time taken to calculate the *TFBI* values of all the links (denoted as *t*_*2*_), and the computation time to determine the actual most critical links from the candidate critical links using the traditional full-scan approach (denoted as *t*_*3*_). *t*_*2*_ mainly includes searching the paths between all OD nodes in the network to calculate link betweenness and estimating the OD matrix. Note that *t*_*1*_ depends on the sample road network size; and *t*_*3*_ is mainly affected by the number of candidate critical links. In this case study, the same sample network is used to calibrate the coefficient *r*. Therefore, the calculation time *t*_*1*_ for different numbers of candidate critical links is the same. Similarly, *t*_*2*_ is the same for different numbers of candidate critical links.

**Table 4:** Computational performance of the *TFBI*-based approach.

*TFBI*-based approach											*NRI*
Number of candidate critical links	50	100	150	200	250	300	350	400	450	500	3,207
**Computation time *t***_***1***_ **(min)**	5	5	5	5	5	5	5	5	5	5	-
**Computation time *t***_***2***_ **(min)**	16	16	16	16	16	16	16	16	16	16	-
**Computation time *t***_***3***_ **(min)**	10	18	27	34	43	51	60	67	73	82	-
**Total computation time**	31	39	48	55	64	72	81	88	94	103	738

The accuracy increases with the number of candidate critical links and the calculation time increases. The ratio of the number of candidate links to the real critical links chosen should be at least six to one. Using the *NRI* for criticality identification, traffic assignment is carried out every time a link is removed and thus must be carried out as many times as the total number of links in the road network. The computation time of the traditional full-scan method based on the *NRI* is 738 min (see Table 4). To achieve 100% accuracy in identifying the 50 most critical links, the minimum computation time of the *TFBI*-based approach (when the number of candidate critical links is 150) is 48 min when using the same equipment as in the traditional full-scan method. This is approximately 6.5% of the computation time required for the traditional full-scan method. If the number of candidate critical links is 300 (six times the given 50 most critical links), the computation time is 72 min, which is approximately 9.8% of the computation time required for the traditional full-scan method. Considering the above results, the proposed *TFBI*-based approach can identify the critical links in a large-scale network and significantly reduce the computational burden compared to that incurred using the traditional full-scan method.

## Conclusions

This article proposes a novel approach based on the *TFBI* to identify critical links in large-scale networks. The *TFBI* is established considering betweenness, link traffic flow, and endpoint OD demand. The systematic indicators computed using the traditional full-scan method can be used to accurately identify critical links. However, in large-scale road networks, it is a computationally intensive to determine the critical links using the traditional full-scan method. In the *TFBI*-based approach, candidate critical links are pre-selected according to their *TFBI* values, and then the preferred number of critical links is determined from the candidate critical links using the traditional full-scan method. The *TFBI*-based approach requires less calculation time compared with the traditional full-scan method to achieve the same accuracy using the same computer equipment. Because the systematic index *NRI* is used to calibrate the coefficient in the *TFBI*, the identification result of the *TFBI*-based approach tends to be more accurate than that of the available link-based indicator. According to the case study, the value of weight coefficient *r* in the *TFBI* and the number of candidate critical links affect the accuracy of the *TFBI*-based approach. The calculation time increases with the number of candidate critical links and the accuracy increases. Regarding the trade-off between accuracy and computational efficiency, an appropriate ratio of the number of candidate critical links to the real critical links is recommended.

The *TFBI* for indirectly evaluating network efficiency loss implies that each link has the same probability of interruption. In an actual road network, link interruptions occur with varying probabilities. Some researchers have focused on vulnerability analysis when considering the probability of interruption and the consequences thereof [38–40]. Future research should thus seek methods for quickly identifying critical links in relation to their probability of experiencing disruption. The proposed method focuses on single-link disruptions that occur more frequently in real life. Identifying the most critical combinations of links among a large number of combinations is a challenging issue. Producing an effective algorithm for identifying the set of critical links deserves more attention in future research [41, 42]. The user equilibrium assignment model is adopted in our study. The user’s path-choosing behaviour affects the identification of critical links. In the future, a traffic flow assignment model considering the user’s response to a failed link should be established for identifying critical links.

The criteria for the sample network were tested based on a partial network in Changchun City, and a generic recommendation in relation to the exact values of the proposed selection criteria will be verified in further research. Further studies are also required to investigate strategies for choosing an appropriate number of candidate critical links.

## Supporting information

S1 File Changchun road network and travel data. (ZIP)
